# Effect of Lower Body Compression Garments on Hemodynamics in Response to Running Session

**DOI:** 10.1155/2014/353040

**Published:** 2014-08-18

**Authors:** Tomas Venckūnas, Eugenijus Trinkūnas, Sigitas Kamandulis, Jonas Poderys, Albinas Grūnovas, Marius Brazaitis

**Affiliations:** Lithuanian Sports University, Sporto Str. 6, 44221 Kaunas, Lithuania

## Abstract

*Purpose*. Compression garments are often worn during exercise and allegedly have ergogenic and/or physiological effects. In this study, we compared hemodynamics and running performance while wearing compression and loose-fit breeches. We hypothesized that in neutral-warm environment compression breeches impair performance by diminishing body cooling via evaporative sweat loss and redistributing blood from active musculature to skin leading to a larger rise in body temperature and prolonging recovery of hemodynamics after exercise. *Methods*. Changes in hemodynamics (leg blood flow, heart rate, and blood pressure during orthoclinostatic test), calf muscle tissue oxygenation, and skin and core temperatures were measured in response to 30 min running (simulation of aerobic training session) followed by maximal 400 m sprint (evaluation of running performance) in recreationally active females (25.1 ± 4.2 yrs; 63.0 ± 8.6 kg) wearing compression or loose-fit breeches in randomized fashion. *Results*. Wearing compression breeches resulted in larger skin temperature rise under the garment during exercise and recovery (by about 1°C, *P* < 0.05; statistical power > 85%), while core temperature dynamics and other measured parameters including circulation, running performance, and sensations were similar compared to wearing loose-fit breeches (*P* > 0.05). *Conclusion*. Compared with loose-fit breeches, compression breeches have neither positive nor negative physiological and performance effects for females running in thermoneutral environment.

## 1. Introduction

Compression garments are elastic tight suits that have long been used to assist venous return and reduce peripheral swelling in vascular patients [1, 2] and healthy humans [3] and to aid in other conditions such as orthostatic intolerance due to dehydration or decrease peripheral muscle pump [4, 5]. Commercially available compression garments of multiple producers are available on the market, engineered not only to snuggly fit the body but also to potentially improve exercise performance [6], and benefits such as improved recovery when worn after endurance [2, 7, 8] or strength exercise [9] have been observed in some while not in all studies [10–13].

Increasing popularity of the use of compression garments during various exercise activities renders investigation of the possible effects these garments may have. While some studies have demonstrated ergogenic effect of the compression garments [14], most of the published research has failed to support any effects of garments for performance capacity in a wide range of exercise tests [2, 13, 15–22]. Those reports that have shown ergogenic effect did not disclose any of the possible underlying mechanisms [14]. However, physiological basis behind the ergogenic effect of compression garments may be related with the improvement in muscle tissue oxygenation [23, 24], and while the mechanisms by which it could manifest remain unclear, it could be associated by increased skin temperature under the garment [25] and ameliorated venous circulation because of the external pressure of the garment [5, 26].

Also, augmented venous return after exercise [1, 26], including during postural changes [27], has been ascribed to compression garments as recovery enhancing effect. Symptoms of orthostatic intolerance may be more prevalent among women [28], and lower body compression garments have been shown to be effective in reducing orthostatic intolerance after the exercise [5]. Also, among the positive effects of the lower body compression garments, an augmented venous return during prolonged standing in women has been reported [29]. At any rate, the modified hemodynamics by the effect of compression garments may comprise modified training stimulus and thus have long term effect if worn regularly during training. Therefore, the effects of compression garments on acute adaptation to exercise are of interest and importance.

The changed hemodynamics (blood flow redistribution) due to wearing special type of garment may be reflected in the changes of other physiological parameters, sensations, and perceived exertion of the exercise and thus have either positive or negative consequences on working capacity, depending on the exercise mode, training status and motivation of the individuals, and environmental conditions. Although compression garments have been shown to neither affect cardiovascular function during orthostatic test in resting conditions [21] nor change cardiovascular response to orthostatic test during recovery after exercise in syncope-prone athletic individuals [5], the effects of compression garments on central and peripheral hemodynamics (cardiovascular regulation of muscle perfusion systemic blood flow) in healthy subjects in response to exercise have not been well investigated. The aim of this study was to evaluate the effect of lower body compression garments on the cardiovascular function in response to running session in thermoneutral environment. We have raised a hypothesis that in these circumstances nonathletic females may have increased thermoregulatory burden leading to steeper rise of internal temperature, augmented cardiovascular response, and consequently diminished performance, as well as prolonged recovery in hemodynamics.

## 2. Methods

### 2.1. Subjects

Thirteen young adult healthy females volunteered for the study. Mean (SD, range) values of the general characteristics of the participants were as follows: age 25.1 (4.2, 19 to 32) years, height 170.2 (6.3, 160 to 180) cm, body mass 63.0 (8.6, 52.2 to 81.0) kg, body mass index 21.9 (2.4, 19.0 to 26.3), and impedance-based estimation of (lower body) fat content 26.5 (6.4, 18.0 to 37.3) percent. All volunteers participated in recreational physical activities two to four times per week and were considered recreationally physically active. The study was given permission of the institutional ethics committee and was conducted consistent with the principles outlined in the Declaration of Helsinki. Prior to the initiation of the study, each of the participants read informed consent form and signed it in agreement to engage in all testing procedures.

### 2.2. Experimental Protocol

Sessions were separated by 7 days and included, in randomized fashion, either loose-fit (control) or experimental lower body compression garments worn throughout the duration of the testing session. Participants abstained from exercise for 2 days and from caffeine and food 2 h before testing and arrived to the laboratory at the same time of the day for both occasions. Running was performed on a 200 m indoor athletic track, and other measurements have been done in the nearby university's laboratory of human physiology. Environmental temperature and relative humidity were stable at 20–22°C and 46–53 percent, respectively, and did not differ between the testing conditions. Participants wore the same shoes, socks, and T-shirts for both sessions and one of the two types of breeches. Each type of the garment was used for the first time during the study, and all participants were given their own set of loose-fit and compression breeches. The participants were acquainted with the experiments and their order before the study.

The subject's nude body mass was measured first. Then the rectal probe was inserted; the subjects dressed up, went to the lab where they were equipped with the measuring devices (HR meter, ECG electrodes, and cuffs around the arm and the calf), and rested there for 10 min on a couch, after which resting subjective thermal sensation, clothing wettedness, shivering/sweating sensation, baseline skin and core temperatures, and resting hemodynamics were recorded, and orthostatic test was performed. Volunteers next performed running session during which HR, temperature, and rate of perceived exertion (RPE) were recorded and time for the last 400 m sprint was measured, and then they were back to the laboratory to undergo the measurements during recovery in the same order as they had been performed at baseline. The duration of entire experimental procedure for one subject was about 120 min per visit. Subjects were not allowed to consume any meal or liquid until all the measurements had been done and the subject's nude body mass was finally measured. The layout of the measurements undertaken during a single visit is presented in Table 1.

### 2.3. Experimental Garments

The compression breeches chosen for the experiment were manufactured of 74% polyamide and 26% elastane (“Compress series” capri pants, Audimas, Lithuania). The elastic reference (control) breeches were manufactured of 91% polyamide and 9% elastane (Audimas, Lithuania). The weight of both types of fabric was 220 g*·*m^−2^, and both were of black color. The breeches reached around the middle of the shank and their size was fitted to participants on the basis of the respective company guidelines involving measures of height, weight, and girth circumferences. The external pressure compression breeches generated on the thigh and the upper calf region were ~17 mmHg and ~18 mmHg, respectively, while control breeches exerted external pressure of less than ~4 mmHg on calf or thigh during standing. These were measured by I-scan system (Evolution, Tekscan Ltd., South Boston, MA, USA), using tactile sensor (Map 9801; Tekscan Ltd., South Boston, MA, USA) and dedicated software (I-scan, Tekscan Ltd., South Boston, MA, USA) for data processing and analysis. Each garment was worn only once, and each subject had her own set of garments for each of the two sessions.

### 2.4. Exercise Protocol

Following preexercise measures, participants individually ran 4 km (20 laps) in the indoor 200 m athletic track led by a custom made red-light leader installed at ~4 m height on the balcony of the arena. The speed was set at 90 seconds per lap, that is, 7 min 30 sec per km pace, and 4 km was covered in 30 min. In 5–10 seconds of the completion of this continuous steady pace submaximal 4 km jog, subjects sprinted for two additional laps (i.e., total 400 m) with maximal efforts to finish the quickest time possible, and the stopwatch was used to measure the times of each of the two laps by the experienced researcher. Subjects were verbally encouraged to exert maximally by using standard phrases at the start of the sprint, then after the first lap, and in the final straight.

#### 2.4.1. Body Mass and Heart Rate

Before and after the testing session, nude mass was measured on electronic scales (TBF-300, Tanita UK Ltd., UK) to estimate the body composition and to evaluate changes in body mass (with accuracy of 0.1 kg). Heart rate (HR) was measured (S-625X, Polar Electro, Kempele, Finland) throughout the testing and then consecutive 5 seconds average HR was used for the analysis.

#### 2.4.2. Leg Blood Flow

Leg blood flow measurement during the passive changing of the calf position was performed by the means of modified Dohn's plethysmograph, as described recently [31]. Air-filled latex rubber measuring conical ring cuff (interior pressure, 4 cm H_2_O; width, 5 cm; upper/lower internal diameter used 110/100, 120/110, 130/120 mm, which was selected according to the individual circumference of the calf) was fitted around the thickest part of the right calf. Before the start of the measurements, subjects were left supine in a quiet laboratory at 22-23°C temperature in a comfortable supine position for 20 min on a custom-made couch devised with a shaft for passive changing of the position of the lower body segments. Then baseline value of the right calf girth was recorded, and quick lowering of the right leg from the heart level by 27 cm (the reference point being a cuff), which corresponds to 20 mmHg increase of the hydrostatic pressure at the segment level [32], was performed to induce the filling phase. After the volume of the calf has stabilized (it took about a minute at baseline and 10 to 60 seconds after the exercise), the leg was quickly raised again to a horizontal position to invoke venous outflow (emptying phase), and the measurement continued until the volume of the calf became stable again (i.e., the inflow and outflow equilibrated, which took about 30 seconds at baseline and 5 to 30 seconds after the exercise). The left leg was kept straight in the horizontal position during the entire period of hemodynamic measurements of the right leg.

Plethysmograph was repeated within 10 min after the termination of running and then after 30 min of recovery. The data were in real-time printed out by an ink-jet recorder throughout the test. Peak arterial blood flow of the leg was calculated from tangent to the initial fast increase in the calf volume curve after its drop below heart level and was expressed in mL of blood per 100 mL of tissue per minute. Peak venous emptying rate of the calf was calculated from tangent to the initial fast slope of the venous volume curve after the raise of the leg to heart level and was expressed in mL of blood per 100 mL of tissue per minute. Venous reserve volume of the calf was defined as an increase in volume of the calf after moving it down to lower position and was expressed in mL per 100 mL of tissue [33].

#### 2.4.3. Orthoclinostatic Test and Near-Infrared Spectroscopy

After 5 min of horizontal lying on a tilt-table (“Veronese,” Italy), the head-up tilt was smoothly performed in 30 seconds to passively change the posture from horizontal to vertical (90 degrees). After 3 minutes in vertical position, the table was tilted back in 30 seconds to horizontal position for another 3 minutes of data recording. During the procedure, ECG was recorded by means of computerized system (“Kaunas-load,” Kaunas, Lithuania). Heart rate averaged every 10 s was used for analysis. Arm cuff arterial blood pressure was measured before the orthostatic test and in one minute intervals during the testing procedure.

Near-infrared tissue spectroscopy (InSpectra; Standard System Model 325, Hutchinson Technology Inc., Hutchinson, MN, USA) was employed to continuously record the oxygen saturation [34, 35] in posterior calf muscles during the orthostatic test at baseline (resting conditions) and 40 minutes after the cessation of exercise (recovery conditions). The sensor for recording of infrared signal was placed over the middle portion of* m. gastrocnemius lateralis* of the right leg. Oxygen saturation readings were recorded continuously every 3.5 s. Optical cable with a probe spacing (i.e., the distance between the probe's sending and receiving fibers) of 25 mm was used. A light scattering calibrator was used to normalize the tissue spectrometer during startup of the system and before each measurement [35]. Averages of the three consecutive readings were used for comparison. Analysis of the data was performed with using absolute values and relative values expressed to baseline values as 100 percent.

#### 2.4.4. Skin and Body Core Temperatures Measurements

Temperatures of body core (*T*_re_) and skin (*T*_sk_) were measured. Measurement of *T*_re_ was performed with a thermocouple (Rectal Probe, Ellab, Hvidovre, Denmark; accuracy 0.01°C) inserted (placed by the subject herself) to a depth of 15 cm past the anal sphincter, and values were recorded at baseline, then at 10 and 20 minutes of running, after the completion of 30 min running, after the 400 m sprint, and then at 5, 10, 20, and 30 minutes in recovery. *T*_sk_ was measured with thermistor taped at lower/distal part of the thigh (DM852, Ellab, Hvidovre, Denmark; accuracy 0.01°C), and values were recorded at baseline, after the completion of running, and then at 5, 10, 20, and 30 minutes in recovery.

#### 2.4.5. Subjective Ratings of Perceptions

The method to measure subjective ratings for the whole body has been described elsewhere [30, 36]. Briefly, ratings of thermal sensation ranged from 1 (very cold) to 9 (very hot), with 5 being neutral. The clothing wettedness sensation was from 1 (dry) to 4 (wet), and shivering/sweating sensation ranged from 1 (vigorously shivering) to 7 (heavily sweating). The scales for each sensation are listed in Table 2. The ratings for thermal sensation, clothing wettedness sensation, and shivering/sweating sensation were reported by the participants at baseline, after the completion of running, and then at 5, 10, 20, and 30 minutes in recovery. Rate of perceived exertion (RPE, Borg scale) was obtained at four time points: at 10 min intervals during the run and after the sprint.

### 2.5. Statistical Analysis

The data were tested for normal distribution using the Kolmogorov-Smirnov test, and all scale data were normally distributed. The data are presented as mean and standard deviation. A repeated-measures (condition × time) analysis of variance (ANOVA) was used to determine differences between the conditions (i.e., test of the garment effect) for the measured variables. Where significant main effect was found, *t*-test was used to determine individual significant differences. For ordinal data the nonparametric Wilcoxon signed-rank test was performed to compare the changes in subjective ratings of perceptions. Significance level was set at *P* < 0.05. Statistical power (SP) was calculated for all the measured indices by using the sample size *n* = 13, average and standard deviation, and alpha level of 0.05.

## 3. Results

### 3.1. Running Capacity and Heart Rate (HR)

The capacity of running the 400 m sprint after the completion of 30 min of steady pace jogging was not affected (*P* > 0.05; SP < 20%) by the type of garment (94.3 (14.6) and 94.1 (13.3) seconds in loose-fit and compression breeches conditions, resp.). Also, pacing strategy (the first 200 m split time) was identical, 44.1 (7.6) and 44.1 (8.6) seconds in loose-fit and compression breeches conditions, respectively.

Resting HR and the dynamics of HR during running and in recovery were almost identical in both conditions (Table 2), and there was no tendency for the compression garment to affect the HR in either direction. In both conditions there was a substantial increase in HR from 15th to 30th of constant pace jogging and further increase to peak HR value during the final spurt (*P* < 0.0001; SP > 99% in all cases). Recovery of HR was again highly similar between conditions (Table 3).

### 3.2. Hemodynamics

There was a reduction of both systolic and diastolic blood pressures after the exercise at all time points before, during, and after the table-tilt test (*P* < 0.01 or more significant; SP > 99%), but the dynamics was not dependent on the type of the garment worn (*P* > 0.05; SP < 30%). During the orthostatic test, tissue oxygenation was the same (it fluctuated around 66 percent on average) in both types of garments before the exercise, while there was a nonsignificant (*P* > 0.05; SP < 25%) tendency for oxygen content to be higher wearing the compression garment as compared with loose-fit breeches (it fluctuated around 70 and 66 percent on average) after the exercise. As compared with loose-fit breeches, there was no effect of the compression garment on HR and systolic and diastolic blood pressures at rest or during the orthostatic test (*P* > 0.05; SP < 35%).

Plethysmographically measured dynamics of the leg circulation parameters were changed dramatically (*P* < 0.001 to *P* < 0.0001; SP > 99%) by the exercise, with highly significant increments in arterial blood flow from baseline up to 30 minutes of recovery and natural concomitant drop in venous reserve volume; on the other hand, venous emptying rate remained unchanged at the measured time point in recovery as compared to baseline (Table 4). Arterial blood flow of the calf was slightly lower (*P* < 0.05; SP > 85%) at baseline, and venous emptying rate was slightly higher (*P* < 0.05; SP > 85%) after 30 min of recovery in compression garment condition as compared with loose-fit breeches, but the difference was absent during the acute phase (within the first 10 minutes) of the recovery after the exercise, when circulation of the leg was several-fold increased (Table 4).

### 3.3. Subjective Ratings and Changes in Body Temperature and Mass

Scores of RPE were 10.2 (1.8), 12.2 (2.3), 14.3 (2.0), and 18.2 (1.6) in compression garment condition and 10.6 (1.6), 12.2 (2.0), 13.8 (2.9), and 17.8 (2.0) in loose-fit garment condition at the 10th, 20th, and 30th minute and at termination of the final 400 m sprint, respectively (*P* > 0.05 between conditions at all time points). Participants lost 0.32 (0.11) and 0.35 (0.13) kg of body mass after the running session in compression and loose-fit garments, respectively (both *P* < 0.0001; SP > 99%; no effect of garment type). Ratings of thermal sensation, sensation of wettedness of clothing, and sweating sensation increased in response to exercise with either type of garment (Table 5) and were not different at any time point between types of breeches (*P* > 0.05).

**Figure 1:**
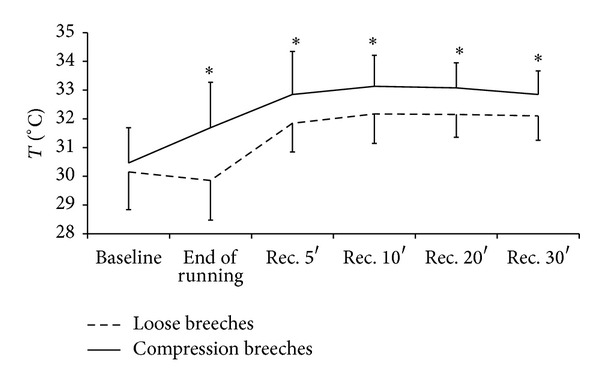
Changes in calf skin temperature (the area covered with the garments) when wearing loose and compression experimental breeches. ^∗^ is indicative of a significant difference at the time point between the conditions (*P* < 0.05). Rec. 5′ to 30′ stand for recovery up to 30 minutes after the running session.

Skin temperature under the garments was increased from baseline moderately in response to exercise and during 30 min of passive recovery in both conditions, with significantly larger increase when wearing compression breeches (Figure 1). Skin temperature was increased from baseline to immediately after exercise in compression garment condition, while it was not until the 5th minute in recovery for the skin temperature to increase above baseline in loose-fit garment condition. Core temperature increased progressively during exercise and reached peak values of 38.8 (0.3) and 38.8 (0.2)°C in compression garment and loose-fit garment conditions, respectively, by the end of running session, with no differences between conditions in either peak values or overall dynamics (*P* > 0.05; SP < 20%).

## 4. Discussion

The main aim of this study was to disclose the effects compression breeches might have on physiological parameters, with the emphasis on hemodynamics, in response to endurance running bout in nonathletic females. With the exception of the increased skin temperature of the undergarment region, no significant differences were found for rate sensations and hemodynamic parameters both at rest and in response to running session. Also, the type of garment (compression compared to loose-fit breeches) did not affect the working capacity by the end of running bout as measured by 400 m of sprinting.

### 4.1. Physiological Responses

Higher skin temperature under the compression garments but identically increased core temperature was recorded in our study as well as other studies [2, 11, 21, 37]. Faster and greater rise of skin temperature has also been reported under compression garments during a warm-up [25]. A higher skin temperature was probably achieved because of the diminished “chimney effect” (lower air circulation and convection) during running in the condition with compression breeches, while loose-fit garments allow more heat dissipation via convection. However, we have not observed that difference in skin temperatures was associated with differences in other physiological parameters, perceptions of the efforts, or improved running performance.

The reduction of nude body mass did not differ between garments, suggesting similar sweat production rate and subsequent loss of body water. This also implies that evaporative cooling was of similar extent and is not impeded by the compression breeches acting as “second skin” in those moderate temperatures and moderate to high intensity running for half an hour conditions. Consistently, the dynamics in core temperature was identical for both conditions. Similar findings were found by other authors who have used running exercise of similar duration and intensity [2], and this was the case in one study even in hot environment [38]. Of note, in both the latter study and our study the average decrease in body mass was lower than one percent; thus, the extrapolation of the effect to more extreme situations such as long runs in warm environment should be avoided. It is important to recognize that caution should be exercised when choosing the type of garment for more prolonged exercise and/or in warmer environment when higher skin temperature and impaired heat dissipation may impose greater physiological strain, psychological perception of the intensity of the exercise, and/or decrease of performance. On the other hand, compression garments may have beneficial effect as a thermal insulator in cool and windy environment or during high speed sports [2, 38].

No significant garment effect on HR at rest and during the exercise was observed in the current study. Our data are in agreement with Berry et al. [39] who have reported no effect of compression tights on HR during close-to-maximal-HR run and Ali et al. [18] who have shown no effect of compression stockings on the HR during 10 km time trial in well trained distance runners. As HR did not change, it could be assumed that cardiac output was also unchanged by wearing elastic compression garments, and it is unlikely that the type of garments could affect venous return or stroke volume in response to running training session. Also, in support to most of the studies that found any ergogenic effects of compression garments, no cardiovascular or metabolic effects of the compression stockings during exercise [40] and no improvement in running economy [19] in endurance athletes were observed.

Although lower body compression garments alleviated orthostatic stress after the exercise in orthostatically intolerant athletes [5], no effect on cardiovascular response to orthostatic stress after exercise in normal females was detected in our study, and it has been shown recently that even whole body compression garments do not change cardiovascular function during orthostatic test at rest [21]. By means of near-infrared spectroscopy Agu et al. [1] reported an increase in leg muscles oxygenation in patients with venous insufficiency when wearing the compression stockings, while our study has not disclosed any benefits of compression breeches on calf muscle oxygenation in healthy females in response to running training session.

### 4.2. Exercise Performance

We have shown no effect of the compression breeches at the final 400 m sprint after the continuous submaximal running in healthy nonathletic women. Ali et al. [18] found no evidence of ergogenic effect in time trial of similar duration in highly trained runners, while the anaerobic power (vertical jump capacity) was better preserved when compression stockings had been used during the run. On the other hand, compression stockings were shown not to affect the fatigue development rate during submaximal static contraction or the recovery of* triceps surae* in nonathletes [41]. Some studies have revealed an increased vertical jumping [24] but not sprint running capacity [2, 24] while wearing lower body compression garments. Compression tights have been reported to improve movement economy in runners at submaximal speeds [42]. It has been proposed to do this by the effect of enhanced circulation and decreased muscle oscillation [42]. However, later study on the effects of compression stockings detected no effect on running economy in athletes [19], and our data on effort, sensations, exercise HR, and core temperature suggest no possible effects of compression breeches on endurance running capacity in healthy females performing in thermoneutral environment.

Overall, the results of the effects of the compression garments are controversial, partly because of the large heterogeneity among studies, including factors such as selection of subject population (including differences in body composition and especially thickness of the subcutaneous fat layer), indices measured, the protocol of the study, the type of the control and experimental garments used (and whether they have been used during or both during and after exercise), and parameters of exercise test applied [6]. As regards the type of compression garment tested, there is some evidence that the effect may not be related to the different length of the lower body suits used. In well-trained endurance athletes, elastic stockings, tights, and whole-body compression suits making different compressive surface were equally found not to change various measured indices of acute adaptation to submaximal and maximal running [16]. Also, different lower body compression attire was demonstrated not to affect male runners' response to 400 m sprint run [22]. In support to our results, it has been concluded by most other researchers that compression garments in general have limited if any ergogenic potential in healthy subjects [2, 6]. However, recent studies in this area have supported earlier reports [23, 29] that rather high external pressure by the lower body compression garments may be required to induce the desirable effects [43, 44]; thus, many of the previous studies in the field may have missed the point by not attaining the minimum threshold level of the pressure to disclaim the sought effects of the compression attire.

As suggested by the producers of the compression breeches, the selected garment sizes based on major anthropometrical characteristics of the subjects indeed generated external pressure ~18 mmHg at the upper calf region and ~17 mmHg at the middle thigh region as measured in our subjects. However, because of the step nature of breech sizes available, we appreciate that the variability of the pressure was rather substantial between the subjects (range, 15 to 21 mmHg for the calf; 16 to 21 mmHg for the thigh region) and that the pressure applied was measured only for resting (standing position) conditions and not during the running activity. However, while the pressures applied by breeches were above the minimal required threshold to manifest the putative effects of lower body compression garment [29], either during or after the running, we cannot eliminate the possibility that even higher pressures would induce favorable ergogenic and/or anaplerotic effects, to the analogy of the recent study which showed that thigh muscle hemodynamic could be affected during the recovery phase after the exercise while wearing compression shorts of twice as large (37 mmHg) an external pressure [43] when compared to required minimal compression strength [18, 45]. On the other hand, these higher pressures exerted by the compression garments would probably be associated with more pronounced unpleasant sensations by the participants [29, 45], and healthy young women who comprised our subject populations thus would unlikely be wearing such attire during/after their leisure time activities even if it comprised some benefits on recovery or working capacity. In addition, the most recent publication in the area also supports moderate (15–20 mmHg) rather than high (25 mmHg) pressure thigh compression if ergogenic effect during running is expected [44].

### 4.3. Perception of Effort and Sensations (Psychological Effects)

It has been proposed that compression garments snuggly fitting the body may alleviate feelings of fatigue and improve proprioception, psychological comfort, motor control, and overall performance [6, 36]. However, compression garments do not change rate of perceived exertion during the repeated sprint running session [2, 38], as it was the case in our study. Also, no effect of compression garments on sweating, comfort, or thermal sensation was detected in runners [38], which is in agreement with our data of recreationally active females, even though the applied exercise protocol induced changes in perceptions of our subjects that were high on all of the scales used.

## 5. Conclusions

In response to running bout in thermoneutral environment, compression breeches did not affect major hemodynamic parameters in nonathletic females, including blood pressure, heart rate, leg blood flow, and tissue oxygenation. While compression breeches increased skin temperature, no effect on running performance, sensations, or modulation of the hemodynamic response to exercise was observed.

## Figures and Tables

**Table 1:** Measurements performed on the subjects during a single visit.

	Baseline	Exercise				Passive recovery						
	40 min	10 min	20 min	30 min	End of 400 m sprint	3 min	5 min	10 min	20 min	30 min	40 min	45 min
Body mass	•											•
Heart rate	Measured throughout the study											
Skin temperature	•				•		•	•	•	•		
Rectal temperature	•	•	•	•	•		•	•	•	•		
Hemodynamics	•					•	•	•		•	•	
Orthostatic probe with ECG and near-infrared spectroscopy	•								•			
Clothing wettedness sensation	•				•		•	•	•	•		
Shivering/sweating sensation	•				•		•	•	•	•		
Thermal sensation	•				•		•	•	•	•		
Rate of perceived exertion		•	•	•	•							

**Table 2:** Scales used for rating of perception (modified from [30]).

Rating	Thermal sensation	Shivering/sweating sensation	Clothing wettedness sensation
1	Very cold	Vigorously shivering	Dry
2	Cold	Moderately shivering	Slightly damp
3	Cool	Slightly shivering	Damp
4	Slightly cool	Not at all	Wet
5	Neutral	Slightly sweating	
6	Slightly warm	Moderately sweating	
7	Warm	Heavily sweating	
8	Hot		
9	Very hot		

**Table 3:** Heart rate (in bpm) response to exercise test in subjects wearing two types of breeches on two separate occasions.

Heart rate	Condition	
	Loose breeches	Compression breeches
At rest	62.8 (10.2)	65.4 (7.4)
1 min after the start of running	128.2 (9.3)	126.2 (7.5)
14–16 min during running	164.2 (15.2)	162.8 (11.4)
28–30 min during running	171.6 (14.6)	170.7 (12.4)
Average (4–30 min of running)	165.7 (14.0)	164.2 (12.2)
Peak (attained during 400 m sprint)	193.5 (10.1)	192.4 (9.1)
At 1 min in recovery	165.9 (12.7)	166.5 (12.7)
At 3 min in recovery	117.2 (14.8)	115.9 (12.0)
At 5 min in recovery	106.8 (12.7)	106.5 (11.3)

No differences were detected at either time point between the conditions (*P* > 0.05).

**Table 4:** Leg blood flow at baseline and after the running in two types of breeches.

Condition	Parameter	Type of garment	
		Loose breeches	Compression breeches
Baseline	Arterial blood flow, mL/100 mL/min	4.56 (1.06)	3.01 (1.41)∗
	Venous reserve volume, mL/100 mL	2.08 (0.57)	1.68 (0.54)
	Venous emptying rate, mL/100 mL/min	42.2 (27.2)	42.3 (27.3)

3 to 10 min after exercise	Arterial blood flow, mL/100 mL/min	17.0 (4.5)	16.9 (6.3)
	Venous reserve volume, mL/100 mL	1.04 (0.33)	1.15 (0.24)
	Venous emptying rate, mL/100 mL/min	29.4 (15.1)	36.5 (16.5)

30 min after exercise	Arterial blood flow, mL/100 mL/min	7.0 (2.2)	7.1 (3.3)
	Venous reserve volume, mL/100 mL	1.24 (0.32)	1.35 (0.40)
	Venous emptying rate, mL/100 mL/min	24.6 (11.1)	45.6 (30.3)∗

∗Significantly (*P* < 0.05) different from loose breeches condition.

**Table 5:** Subjective ratings of perceptions in response to running in loose and compression breeches.

Perceptions	Time point	Type of garment	
		Loose breeches	Compression breeches
Thermal sensation	Baseline	4.9 (0.9)	4.9 (1.0)
	End of running	8.0 (0.6)	8.2 (0.6)
	Rec. 5′	7.1 (0.8)	7.3 (0.9)
	Rec. 10′	6.5 (0.7)	6.2 (0.8)
	Rec. 20′	5.6 (1.0)	5.5 (0.8)
	Rec. 30′	5.1 (0.8)	5.2 (0.7)

Shivering/sweating sensation	Baseline	3.8 (0.4)	3.6 (0.5)
	End of running	5.9 (0.5)	6.0 (0.6)
	Rec. 5′	5.2 (0.4)	5.4 (0.5)
	Rec. 10′	4.9 (0.3)	4.9 (0.5)
	Rec. 20′	4.2 (0.4)	4.2 (0.4)
	Rec. 30′	4.0 (0.0)	4.1 (0.3)

Clothing wettedness sensation	Baseline	1.0 (0.0)	1.0 (0.0)
	End of running	3.1 (0.8)	2.8 (0.6)
	Rec. 5′	2.5 (0.8)	2.8 (0.7)
	Rec. 10′	2.2 (0.6)	2.2 (0.4)
	Rec. 20′	1.5 (0.8)	1.6 (0.5)
	Rec. 30′	1.0 (0.0)	1.2 (0.4)

Rec.: recovery.

No differences were detected at either time point between the conditions (*P* > 0.05).

## Abbreviations

HR: Heart rate
RPE: Rate of perceived exertion
*T*_re_: Core temperature
*T*_sk_: Skin temperature.
